# Cerebellar Infarction Following Pulseless Electrical Activity Arrest in Advanced Heart Failure With Reduced Ejection Fraction: A Post-resuscitation Diagnostic Pitfall

**DOI:** 10.7759/cureus.103092

**Published:** 2026-02-06

**Authors:** Cristina Suarez Chiriboga, Saketh Jayanthi, Amber Jin, Iheoma Duruiheoma, Roxana Lazarescu

**Affiliations:** 1 Internal Medicine, Wyckoff Heights Medical Center, New York, USA; 2 Internal Medicine, Touro College of Osteopathic Medicine, New York, USA; 3 Internal Medicine, Xavier University School of Medicine, New York, USA

**Keywords:** cardiogenic shock, cerebellar infarction, cocaine-associated cerebrovascular disease, goals-of-care decision-making, heart failure with reduced ejection fraction, hypoxic-ischemic brain injury, neuroprognostication, post-cardiac arrest encephalopathy, post-resuscitation neuroimaging, pulseless electrical activity arrest

## Abstract

Neurologic deterioration following cardiac arrest is frequently attributed to global hypoxic-ischemic brain injury. However, focal ischemic stroke may occur concurrently and represents an important diagnostic pitfall in post-resuscitation care. We report the case of a 59-year-old woman with end-stage heart failure with reduced ejection fraction who developed acute neurologic impairment following prolonged pulseless electrical activity arrest. Early non-contrast CT of the head demonstrated a focal left cerebellar infarction without diffuse cerebral edema. MRI of the brain performed four days later showed no acute intracranial abnormality, illustrating radiographic discordance between early CT and delayed MRI in posterior circulation ischemia. These complementary imaging findings informed ongoing neurologic assessment and multidisciplinary discussions. This case underscores the importance of early neuroimaging and maintaining a broad neurologic differential diagnosis in post-cardiac arrest patients.

## Introduction

Post-cardiac arrest encephalopathy is commonly attributed to global hypoxic-ischemic brain injury, particularly following prolonged resuscitation, as discussed in contemporary post-cardiac arrest care guidelines by Hirsch et al. [1]. However, neurologic injury after cardiac arrest encompasses a heterogeneous spectrum of processes extending beyond diffuse anoxic injury and including focal ischemic lesions, as reviewed by Cronberg et al. [2].

Early neurologic examination is frequently limited in comatose or sedated patients, and premature attribution of neurologic decline to irreversible hypoxic-ischemic injury may lead to uncertain prognostication and challenging goals-of-care discussions, as emphasized in Neurocritical Care Society guidance by Rajajee et al. [3]. Supporting this, Wallin et al. demonstrated that acute focal brain lesions are commonly identified in survivors of cardiac arrest and may be clinically indistinguishable from global hypoxic-ischemic injury when early neuroimaging is not obtained [4].

Substance-related cardiovascular toxicity further increases cerebrovascular risk in critically ill patients. As highlighted by Wilkins, cocaine exposure is associated with vasoconstriction, arrhythmogenesis, and acute cardiac decompensation, all of which may predispose to focal cerebral ischemia [5]. In parallel, Powers et al. emphasized the importance of timely neuroimaging for accurate diagnosis and management of ischemic stroke, even in complex post-cardiac arrest settings [6].

Posterior circulation infarcts present unique diagnostic challenges. False-negative diffusion-weighted MRI in acute stroke was first described by Oppenheim et al. [7], and these limitations have been further synthesized in a systematic review by Edlow et al., highlighting technical constraints and lesion size effects in posterior fossa imaging [8]. We present a case illustrating CT-MRI discordance in posterior circulation ischemia and its relevance to neurologic assessment and care planning.

## Case presentation

A 59-year-old woman with advanced heart failure with reduced ejection fraction (approximately 10%), coronary artery disease, chronic obstructive pulmonary disease, and human immunodeficiency virus (HIV) infection on antiretroviral therapy presented with acute hypoxic respiratory failure due to decompensated systolic heart failure. Although her HIV viral load was undetectable, immunologic recovery was incomplete, with the most recent available CD4 count of 136 cells/µL (July 2025), remaining in the acquired immunodeficiency syndrome range. Persistent immune activation and endothelial dysfunction in treated HIV have been associated with elevated cerebrovascular risk, as reviewed by Hunt [9].

She had a history of cocaine use disorder, with repeated urine toxicology positivity documented from November 2024 through October 2025, suggesting ongoing cocaine exposure. Cocaine-associated vasoconstriction and arrhythmogenesis are known contributors to cerebrovascular and cardiac instability, as described by Wilkins [5].

On presentation, she demonstrated severe anasarca, bilateral pleural effusions, and markedly elevated B-type natriuretic peptide (2,107 pg/mL). During hospitalization, she became increasingly agitated and repeatedly removed oxygen support and refused non-invasive ventilation, likely reflecting evolving hypoxia and/or hypercapnia in the setting of acute decompensated heart failure rather than primary neurologic injury, consistent with post-cardiac arrest encephalopathy frameworks outlined by Hirsch et al. [1].

In October 2025, the patient was found pulseless with pulseless electrical activity. Advanced cardiac life support was initiated, with return of spontaneous circulation after approximately 16 minutes. She was subsequently intubated, an emergent femoral central line was placed, and norepinephrine was initiated for hypotension. She was transferred to the intensive care unit in cardiogenic shock requiring norepinephrine and milrinone.

Later on, neurologic examination off sedation demonstrated lethargy, inability to follow commands, minimal pupillary reactivity, preserved brainstem reflexes, and withdrawal to noxious stimulation without purposeful movement. Electroencephalography demonstrated moderate generalized slowing without epileptiform activity, consistent with indeterminate prognostic patterns described by Rajajee et al. [3].

**Figure 1:**
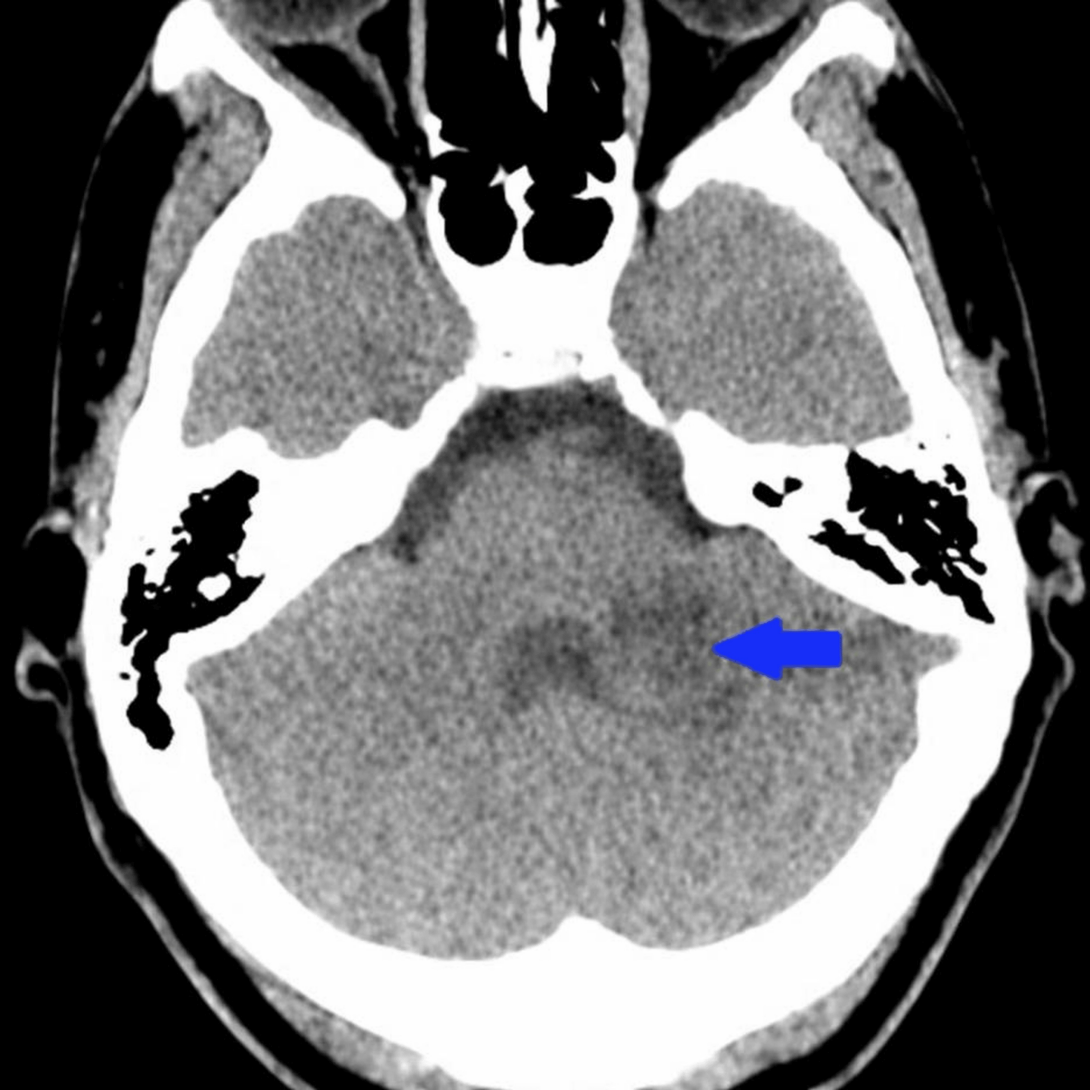
Non-contrast axial CT of the brain obtained in October 2025, performed nine hours after return of spontaneous circulation, demonstrates an approximately 3-cm hypodense lesion in the left cerebellar hemisphere (arrow), consistent with acute to subacute infarction, without hydrocephalus or significant mass effect.

Non-contrast CT of the head performed approximately nine hours after return of spontaneous circulation demonstrated an approximately 3-cm hypodense lesion in the left cerebellar hemisphere consistent with acute to subacute infarction, without diffuse cerebral edema (Figure 1), consistent with early focal lesion detection described by Wallin et al. [4].

CT angiography of the head and neck demonstrated patent posterior circulation without large-vessel occlusion, consistent with diagnostic pathways outlined by Powers et al. [6].

**Figure 2:**
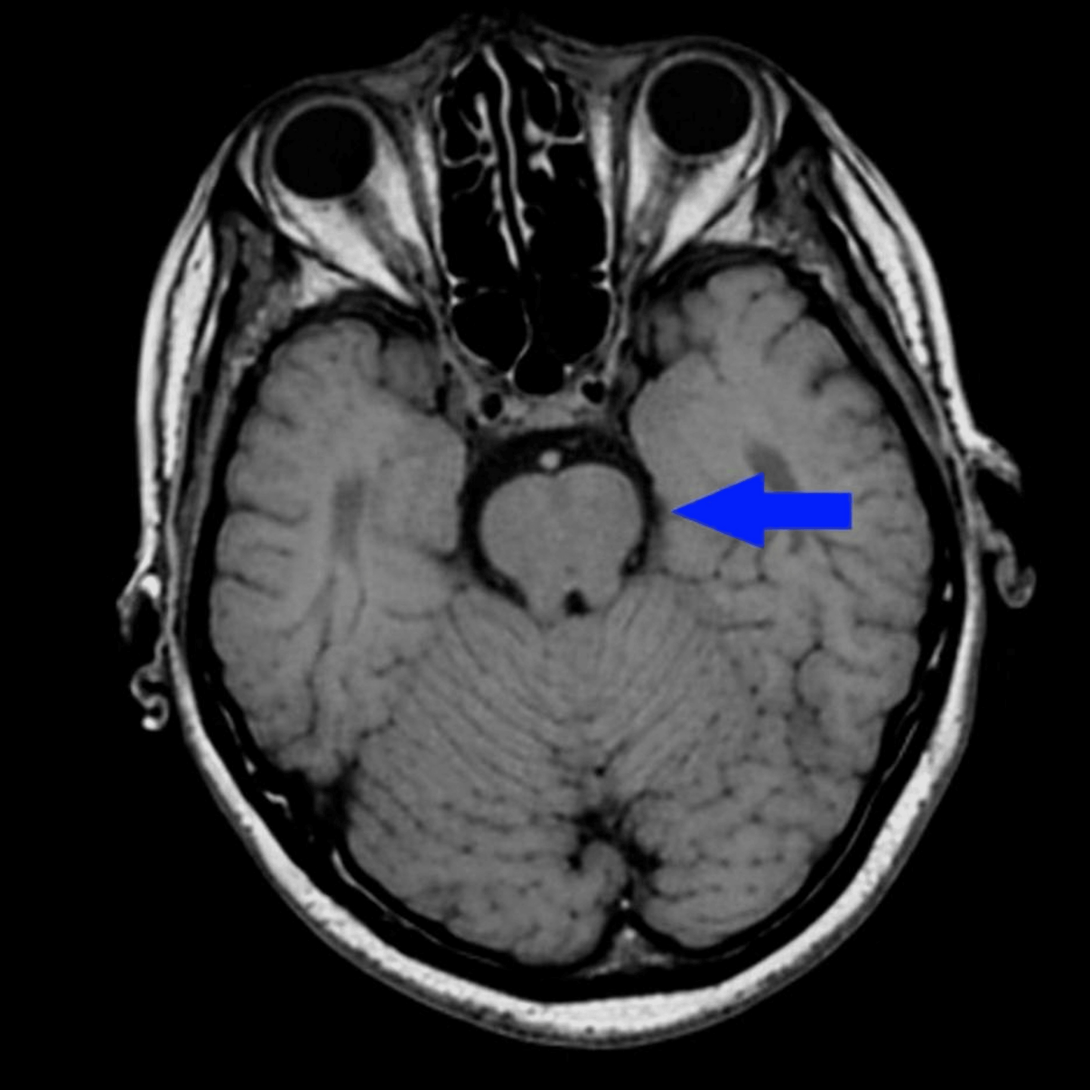
Brain imaging performed approximately four days after return of spontaneous circulation, after hemodynamic stability permitted transport, demonstrates no acute diffusion restriction. The blue arrow corresponds to the same left cerebellar region previously identified on CT (Figure 1), where no corresponding lesion is visualized on follow-up imaging.

MRI of the brain without contrast was performed approximately four days after return of spontaneous circulation, once hemodynamic stability permitted safe transport, and demonstrated no acute diffusion restriction. Imaging demonstrated no acute territorial infarct or hypoxic-ischemic injury (Figure 2). Technical limitations of posterior fossa MRI and timing-related diffusion normalization have been described by Oppenheim et al. and Edlow et al. [7,8].

These findings were incorporated into multidisciplinary discussions with the family in accordance with neuroprognostication principles outlined by Rajajee et al. [3]. Despite maximal supportive care, the patient’s clinical course was dominated by refractory cardiogenic shock and multisystem organ failure.

## Discussion

This case highlights a diagnostic pitfall in post-resuscitation care: reflexive attribution of neurologic deterioration to global hypoxic-ischemic injury, as cautioned by Hirsch et al. and Cronberg et al. [1,2]. Early CT demonstrated focal cerebellar infarction, while delayed MRI showed no acute infarct, illustrating posterior circulation imaging discordance described by Oppenheim et al. and Edlow et al. [7,8]. The CT characterization of “acute to subacute” infarction raises the possibility that diffusion abnormalities had begun to normalize by the time MRI was obtained.

Isolated cerebellar infarction typically does not fully account for profound encephalopathy; in this case, neurologic impairment was multifactorial, occurring in the setting of hypoxia, prolonged low-flow state during pulseless electrical activity arrest, cardiogenic shock, metabolic derangements, and sedation, consistent with heterogeneous brain injury mechanisms reviewed by Cronberg et al. [2].

Multiple factors plausibly contributed to focal ischemia, including systemic hypoperfusion, cocaine-related vasoconstriction, and arrhythmogenesis, as described by Wilkins [5], and persistent immune dysfunction despite virologic suppression of HIV, as reviewed by Hunt [9].

Early identification of focal infarction supported comprehensive neurologic evaluation and avoided premature prognostication, in alignment with guidance from Rajajee et al. [3] and stroke imaging recommendations from Powers et al. [6].

## Conclusions

Neurologic impairment following cardiac arrest may result from both global hypoxic-ischemic injury and concurrent focal ischemic stroke. Early CT imaging can facilitate the identification of posterior circulation infarction that may not be visualized on subsequent MRI. In critically ill patients, however, the timing of advanced neuroimaging may be constrained by hemodynamic instability and vasopressor dependence, as in the present case. Maintaining a broad neurologic differential while balancing the need for early imaging with physiologic stability supports accurate clinical assessment, appropriate prognostication, and multidisciplinary care planning.

## References

[1] Hirsch KG, Amorim E, Coppler PJ (2025). Part 11: Post-Cardiac Arrest Care: 2025 American Heart Association Guidelines for Cardiopulmonary Resuscitation and Emergency Cardiovascular Care. Circulation.

[2] Cronberg T, Greer DM, Lilja G, Moulaert V, Swindell P, Rossetti AO (2020). Brain injury after cardiac arrest: from prognostication of comatose patients to rehabilitation. Lancet Neurol.

[3] Rajajee V, Muehlschlegel SM, Wartenberg KE (2023). Guidelines for neuroprognostication in comatose adult survivors of cardiac arrest. Neurocrit Care.

[4] Wallin E, Larsson IM, Kristofferzon ML, Larsson EM, Raininko R, Rubertsson S (2018). Acute brain lesions on magnetic resonance imaging in relation to neurological outcome after cardiac arrest. Acta Anaesthesiol Scand.

[5] Wilkins JN (1992). Brain, lung, and cardiovascular interactions with cocaine and cocaine-induced catecholamine effects. J Addict Dis.

[6] Powers WJ, Rabinstein AA, Ackerson T (2019). Guidelines for the early management of patients with acute ischemic stroke: 2019 update to the 2018 guidelines for the early management of acute ischemic stroke: a guideline for healthcare professionals from the American Heart Association/American Stroke Association. Stroke.

[7] Oppenheim C, Stanescu R, Dormont D (2000). False-negative diffusion-weighted MR findings in acute ischemic stroke. AJNR Am J Neuroradiol.

[8] Edlow BL, Hurwitz S, Edlow JA (2017). Diagnosis of DWI-negative acute ischemic stroke: a meta-analysis. Neurology.

[9] Hunt PW (2012). HIV and inflammation: mechanisms and consequences. Curr HIV/AIDS Rep.

